# Effectiveness and Safety of Esketamine Nasal Spray in Treatment-Resistant Depression: Early Outcomes from Poland’s First Psychiatric Drug Program B.147

**DOI:** 10.1192/j.eurpsy.2026.10418

**Published:** 2026-07-31

**Authors:** O. Płaza, R. Madejek, M. M. Dutsch-Wicherek, M. Mikulski, A. Szulc

**Affiliations:** 1 Department of Psychiatry; 2 Department of Community Psychiatry, Faculty of Health Sciences, Medical University od Warsaw; 3 Cell Biophysics Laboratory, Nencki Institute of Experimental Biology of the Polish Academy of Sciences, Warsaw; 4 2AB; 5 2DE, Mazovian Specialist Health Centre, Pruszków, Poland

## Abstract

**Introduction:**

Treatment-resistant depression (TRD), which affects about 30% of patients with major depressive disorder, emerges when patients do not respond adequately to at least two antidepressant trials of proper dose and duration. The EMA approved esketamine nasal spray, the first registered psychedelic medication, in 2019 for TRD treatment. In July 2023, Poland launched B.147, the country’s first psychiatric drug program, which grants TRD patients access to esketamine in specialized centers. Poland is also the first EU country to reimburse esketamine treatment for this patient group.

**Objectives:**

To evaluate the real-world effectiveness, safety, and healthcare utilization patterns of esketamine nasal spray in TRD patients treated under Poland’s Drug Program B.147 since its implementation.

**Methods:**

A retrospective registry analysis was conducted at one psychiatric center involved in Drug Program B.147, covering patients from July 2023 to August 2025. Adults with TRD (≥18 years), who failed at least two adequate antidepressant trials, were using an SSRI/SNRI, had no addiction or excluded psychiatric disorders, and could not receive electroconvulsive therapy, were included. Outcomes were clinical response (≥50% reduction in MADRS at 4 weeks), remission (MADRS ≤10), time to response, discontinuation, adverse events, and healthcare utilization. Analysis used mixed-effects models and survival methods.

**Results:**

55 patients underwent the qualification process, with 23 patients meeting criteria to be enrolled in the program (mean age ±41.9 years; 14 female, 8 male, with one female patient being enrolled into the program twice). Baseline MADRS score was ±38 points (severe depression). At 4 weeks, 73.9% (n=17) achieved sufficient clinical response, with 17.6% (n=3) achieving remission. Remission rates improved to 47% (n=8) at 8 weeks. Out of all the patients that finished the entire course of treatment (n=8), 50% (n=4) remained in complete remission. Treatment discontinuation occurred in 8.6% (n=2) of patients by 6 months, due to adverse events (50%; n=1) and lack of efficacy (50%; n=1). Most common adverse events were dizziness (91.3%; n=21), nausea (78.2%; n=18), and dissociation (56.5%; n=13), generally mild-to-moderate and transient, with their frequency and intensity decreasing with treatment time. Serious adverse events were not observed in any of the patients.

**Conclusions:**

Esketamine nasal spray demonstrated clinically meaningful effectiveness in Polish TRD patients under real-world conditions, with response rates comparable to clinical trials. The favorable safety profile and reduced healthcare utilization support the value of Drug Program B.147. These findings provide important evidence for healthcare policy decisions regarding esketamine accessibility in European healthcare systems.

**Disclosure of Interest:**

None Declared

